# Innovations in Mentorship: Implementation of a Mentorship Program in Psychiatry That Encourages Reflection on Intersectionality and Wellness

**DOI:** 10.1192/bjo.2022.119

**Published:** 2022-06-20

**Authors:** Shaheen Darani, Mary Jane Esplen, Certina Ho, Krista Lanctot, John Teshima, Danica Kwong, Jiahui Wong

**Affiliations:** University of Toronto, Toronto, Canada

## Abstract

**Aims:**

Mentorship supports professional development, academic outcomes, and wellness. Effective mentorship can develop careers of faculty through greater access, and equity, diversity, and inclusion (EDI). At a Department of Psychiatry in Canada, a recent survey showed more than 60% faculty were without mentors and would like to have one; and 75% mentors received no training nor resources to support mentorship. The aims of the Psychiatry Mentorship Program are to facilitate sharing of expertise, self-reflection, and career growth among faculty.

**Methods:**

A Mentorship Working Group was formed in 2020–2021. The Mentorship Program design was evidence-informed by a literature review and consultation with other mentorship programs. While a traditional primary mentor-mentee relationship is at the core of the program, the mentorship dyad will be further supported by mentorship groups focused on academic roles, areas of scholarship and career development (e.g., clinician scientists; wellness) or specific groups (e.g., members of a minority group). The program offers an online mentor/mentee matching process, based on faculty self-reported scholarship interests, academic roles, and preferences related to social identity. A three-year evaluation strategy, guided by a logic model, is integrated throughout program implementation. Mentees and mentors are expected to complete a baseline assessment upon program enrolment and annual follow-up questionnaires. Continuous quality improvement of the Mentorship Program will be based on user experience collected via focus groups and interviews where perception and concepts, such as intersectionality, wellness, and EDI, will be explored.

**Results:**

The Mentorship Program pilot was launched in fall 2021 with mentor and mentee virtual orientation workshops offering best practices and opportunities for reflection on challenges that may be encountered during a mentoring relationship. Thirty-six faculty mentors and 60 newly appointed faculty mentees attended the orientation workshops respectively. Workshop evaluations were positive. For example, 93% participating mentors indicated that the workshop met its learning objectives; 80% rated the workshop as excellent. Eighty-seven percent of mentor participants reported increased awareness of best practices to support successful mentorship, including the use of contracts and developmental plans, and indicated the workshop stimulated reflection and learning.

**Conclusion:**

This preliminary positive feedback suggests faculty found the orientation workshops on mentorship to be useful and thus represents an effective mode of facilitating implementation of a department wide mentorship program. We anticipate the implementation of our mentorship program could be adapted to other academic settings.

